# Effects of ultrasound-guided paravertebral block on MMP-9 and postoperative pain in patients undergoing VATS lobectomy: a randomized, controlled clinical trial

**DOI:** 10.1186/s12871-020-00976-1

**Published:** 2020-03-06

**Authors:** Haichen Chu, He Dong, Yongjie Wang, Zejun Niu

**Affiliations:** 1 Department of Anesthesiology, The Affiliated Hospital of Qingdao University, No.16 Jiangsu Road, Shinan District, Qingdao, China; 2 Department of Thoracic Surgery, The Affiliated Hospital of Qingdao University, Qingdao, China

**Keywords:** Video-assisted thoracoscopic lobectomy, Paravertebral anesthesia, Pain, Operative, Matrix metalloproteinase-9

## Abstract

**Background:**

Local anesthesia can reduce the response to surgical stress and decrease the consumption of opioids, which may reduce immunosuppression and potentially delay postoperative tumor recurrence. We compared paravertebral block (PVB) combined with general anesthesia (GA) and general anesthesia regarding their effects on postoperative pain and matrix metalloproteinase-9 (MMP-9) after video-assisted thoracoscopic surgery (VATS) lobectomy.

**Methods:**

54 patients undergoing elective VATS lobectomy at a single tertiary care, teaching hospital located in Qingdao between May 2, 2018 and Sep 28, 2018 were randomised by computer to either paravertebral block combined with general anesthesia or general anesthesia. The primary outcomes were pain scores at rest and on cough at 1, 4, 24, and 48 h after surgery. The secondary outcome were plasma concentrations of MMP-9, complications, and length of postoperative hospital stay.

**Results:**

75 were enrolled to the study, of whom 21 were excluded before surgery. We analyzed lobectomy patients undergoing paravertebral block combined with general anesthesia (*n* = 25) or general anesthesia (*n* = 24). Both groups were similar regarding baseline characteristics. Pain scores at rest at 4 h and 24 h, on cough at 4 h were lower in PVB/GA group, compared with GA group (*P* < 0.05). There were no difference in pain scores at rest at 1 h, 48 h and on cough at 1 h, 24 h, and 48 h between groups. Patients in the PVB/GA group showed a greater decrease in plasma MMP-9 level at T1 and T2 after VATS lobectomy (*P* < 0.05). Postoperative complications and length of stay did not differ by anesthetic technique.

**Conclusions:**

The paravertebral block/general anesthesia can provide statistically better pain relief and attenuate MMP-9 response to surgery and after VATS lobectomy. This technique may be beneficial for patients to recover rapidly after lung surgery and reduce postoperative tumor recurrence.

**Trial registration:**

Chinese Clinical Trial registration number ChiCTR1800016379. Registered 28 May 2018.

## Background

In recent decades, lung cancer is the most common malignant tumor from the worldwide. The most common type of lung cancer that causes death is non-small cell lung cancer (NSCLC), which has caused serious burden on patients and society [1]. Surgery is still the effective treatment for lung cancer. Even if the tumors are complete resected including systemic lymph node dissection, the chance of tumor recurrence is still high due to undetected micro-metastasis [2–4]. Although general anesthesia is the most commonly used anesthetic method for lung cancer surgery, it has higher levels of inflammation and stronger immunosuppressive effects in comparison with regional anesthesia [5]. Nerve block anesthesia such as paravertebral nerve block has many advantages such as reducing opioids and general anesthetic consumption, reducing the inflammatory response and immunosuppression caused by surgical trauma, and can improve the long-term survival rate of postoperative patients with lung cancer [6].

The matrix metalloproteinase (MMP) family plays an important role in tumor recurrence. MMP-9 is the most detected in a variety of malignant tissues and is associated with tumor metastasis and recurrence potential [7]. Immunohistochemical expression and increased plasma levels of MMP-9 have been demonstrated in NSCLC patients [8]. However, to the best of our knowledge, a comparision of the effects of ultrasound-guided paravertebral block combined with general anesthesia and general anesthesia on postoperative pain scores and MMP-9 in VATS lobectomy is rare. Therefore, we compared the effect of PVB/general anesthesia and general anesthesia on pain scores, MMP-9, postoperative complications and length of stay after VATS lobectomy.

## Methods

The study was approved by the Medical Ethics Committee of the Affiliated Hospital of Qingdao University and was performed between May 2018 and Sep 2018. Informed written consent was signed by every patient prior to enrollment in this study. Our study was registered with Chinese Clinical Trial Registry (ChiCTR1800016379). All study procedures were completed at the affiliated hospital of Qingdao university, a tertiary care, teaching hospital located in Qingdao, China. The surgical procedures performed included VATS lobectomy and systematic mediastinal lymphadenectomy.

The inclusion criteria included the following: patients with lung tumors who were undergoing VATS lobectomy, aged 18–70 years, of both genders, American Society of Anesthesiologists physiological statusIto III. The exclusion criteria were used: body mass index ≥30 kg/m^2^, anatomical abnormalities of the thoracic spine identified by chest computed tomography, spontaneous pneumothorax in the medical history, known allergy or hypersensitivity against amino-amide local anesthetics (LA), use of nonsteroidal anti-inflammatory drugs 2 weeks before surgery, coagulopathies in the medical history. Seventy-five patients scheduled for VATS lobectomy completed. 54 patients undergoing elective VATS lobectomy were randomized by computer to either PVB/GA (*n* = 27) or GA (n = 27). Two PVB patients who was with failed PVB and converted to open surgery did not participate in the final analysis. Three GA patients dropped out after randomization. We finally analyzed patients undergoing PVB/GA (*n* = 25) or GA (*n* = 24). Perioperative data were collected by anesthesia personnel (residents, nurse anesthetists and attendings).

### Thoracic paravertebral block technique

We performed ultrasound-guided two-shot paravertebral blocks with 20 ml of 0.375% ropivacaine (AstraZeneca AB, PS05070, Sweden) at the thoracic interspace T4–5 and T7–8. We used long-axis (transverse approach) in-plane techniques for thoracic paravertebral nerve block. Using the ultrasound system (SonoSite M-Turbo, SonoSite Inc., Bothell, WA) to determine the thoracic paravertebral space (TPVS) of T4 and T7 levels in the lateral position, we visualized that the needle tip (Stimuplex D Plus, 0.71 × 80 mm, 22G × 3 1/8,” B.Braun Melsungen AG, Germany) was between the superior costotransverse ligament and the pleura and placed it inside the TPVS, 20 ml of 0.375% ropivacaine (each injection point) was administered after negative aspiration under direct ultrasound imaging [9].

### Intraoperative and postoperative management

On the day of surgery, investigators generated the randomization sequence using a computerized program. The allocation was concealed until shortly before anesthesia. An anesthesiologist who was not involved in this study placed the assignment numbers in opaque sealed envelopes to conceal the randomization sequence. All cases were allocated at random to one of two group: a control group (group GA), receiving general anesthesia and postoperative patient-controlled intravenous analgesia (PCIA), and a treatment group (group PVB), receiving thoracic paravertebral anesthesia combined general anesthesia and postoperative PCIA. Medical Ethics Committee approval and written informed consent were obtained. All patients were conducted by same anesthesiologist who had considerable prior experience with use of PVB.

In both groups, induction of anesthesia was performed with propofol (1–2 mg/kg), sufentanil (0.4–0.5 μg/kg), and cisatracurium (0.15–0.2 mg/kg) for muscle paralysis. After tracheal intubation, maintenance of anesthesia was performed with sevoflurane (1%) in a mixed oxygen/air fresh gas, and cisatracurium as needed in both groups. Analgesia was assured by the ropivacaine solution (0.375%) in the PVB group and by sufentanil as needed in the GA group.

Flurbiprofen 50 mg was intravenous injection at 30 min before the end of surgery in the both groups. When the surgery is finished, all patients were transferred to the postanesthesia care unit (PACU). All patients who were awake were connected with the PCIA pump with sufentanil and ondansetron. Sufentanil was inserted with 1-2 μg/h. A bolus of 2 mL was allowed at every 15 min up to a maximal dose of 10 μg/h.

All patients were treated with IV flurbiprofen in 50–100 mg increments for a Visual Analog Scale (VAS) score of 4/10 or greater or patient request for analgesia. Patients were monitored in the PACU until they met discharge criteria.

### Outcomes

Our primary end point was pain scores at rest and on cough. A VAS was used to assess pain intensity at 1, 4, 24 and 48 h after completion of surgery. The secondary outcomes were plasma concentrations of MMP-9, postoperative complications and postoperative hospital stay. Postoperative complications including pneumonia, atelectasis, air leak, atrial fibrillation, hypotension and postoperative nausea and vomiting (PONV).

Blood samples were obtained 10 min before anesthesia (T0), at the end of surgery (T1), and at 12 h after operation (T2). Blood was collected into EDTA tubes and centrifuged at 4000 g for 15 min at 4 °C immediately after sampling. Thereafter, plasma was stored at − 70 °C until all the samples were collected. Plasma concentrations of MMP-9 were measured with commercially quantitative sandwich ELISA kits (Wuhan USCN Business Co., Ltd., Wuhan, China). Standards were prepared, and the appropriate volume of sample or standard was added to a 96-well polystyrene microtitre plate, and incubated for 1 h at 37 °C. Unbound material was removed. Detection Reagent A (biotin-conjugated antibody specific to target protein) was added to each well, and the incubation was continued for 37 °C. After washing with wash buffer 3 times, Detection Reagent B (avidin conjugated HRP) was added to each well, and the incubation was continued for 0.5 h at 37 °C. After washing with wash buffer 5 times, TMB substrate was added to each well, and the incubation was continued for 10–20 min at 37 °C. Once 50 μl stop solution was added to each well, and the absorbance at 450 nm was measured.

Seven known concentrations, ranging from 0.156 to 10 ng/ml was measured for MMP-9. Samples values was used for further statistical analysis. The concentration of target protein in the samples is then determined by comparing the O.D. of the sample to the standard curve.

Demographic information (age, sex, body mass index, and the American Society of Anesthesiologists grade) and pertinent surgical information (operation time, estimated blood loss, type of surgery, histology and stage of tumor) were recorded.

Prospectively collected data included pain scores at 1, 4, 24 and 48 h after completion of surgery, complications (pneumonia, atelectasis, air leak, atrial fibrillation, hypotension, PONV), and length of stay. Both groups received PCA using a mixture of 1 μg/mL sufentanil and 0.08 mg/mL ondansetron with the pump set to deliver doses of 1-2 μg/h intravenous sufentanil with a 15-min lockout time. If the VAS score is greater than 3, 50–100 mg of flurbiprofen was injected intravenously. Nausea and vomiting were treated with intravenous 8 mg ondansetron. Ambulation early after VATS lobectomy was a postoperative ERAS element. The patients were made to walk along the bedside, if possible, walk around the ward always accompanied by family member and the nursing staff on the following day after surgery. Oral liquid on the first day after surgery, and a semi-liquid diet after flatus passage were started at postoperative day 1. The early postoperative intake of solids was initiated at postoperative passage of flatus. All patients were subjected to enforced early mobilization. Perioperative management was similar in both groups.

### Statistical analysis

The sample size calculation was based on mean VAS scores (2.53 ± 0.83) from our hospital in the pilot study. To have a greater than 90% power with an overall 2-sided typeIerror rate of 5%, and consider withdrawal and loss of follow-up (cases of 10%), at least 22 patients were required in each group.

Continuous variables were expressed as the mean (± 1 standard deviation) or median (95% confidence interval (CI)) when data were not normally distributed and were compared between the two groups using the Mann-Whitney U test. *P* < 0.05 was considered significant for all data. Data were analyzed by use of the statistical package for the social sciences (SPSS 23.0).

## Results

**Fig. 1:**
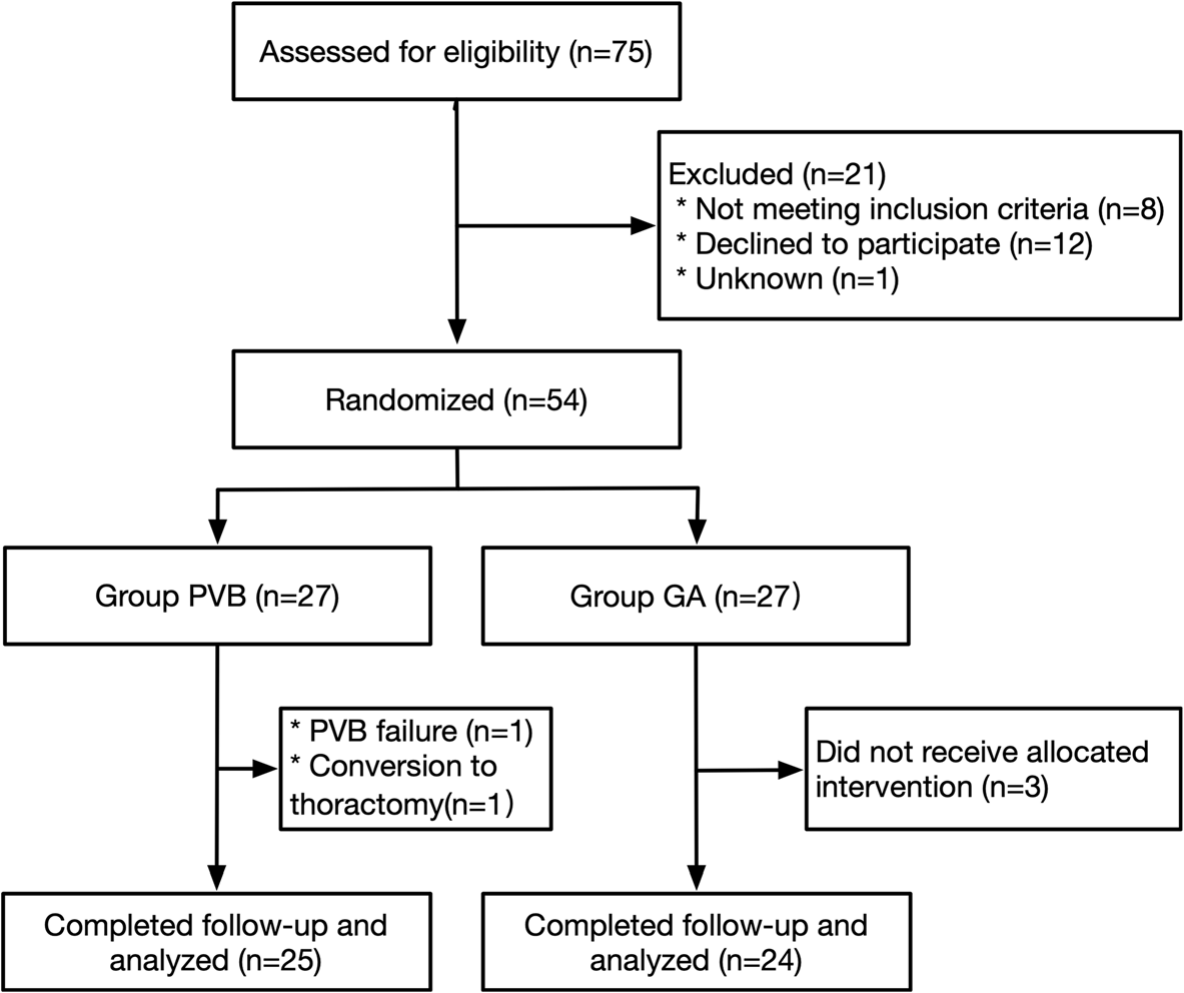
Protocol for patient enrolment in the study groups. Randomized controlled trial comparing PVB/GA versus GA for VATS lobectomy. PVB = paravertebral block; GA = general anesthesia; VATS = video-assisted thoracoscopic surgery

Between May 2, 2018 and September 28, 2018, 75 consecutive patients were assessed for eligibility. Twenty-one patients did not meet the inclusion criteria or refused to participate. The remaining 54 patients provided written consent to participate and were randomized to either group PVB (*n* = 27) or group GA (n = 27). Two PVB patients who was with failed PVB and converted to open surgery did not participate in the final analysis. Three GA patients dropped out after randomization. Final analysis compared therefore 25 PVB patients with 24 GA patients (Fig. 1). All subjects were included in the primary outcome analysis. There were no clinically significant differences in demographic data and surgical data between groups, except for the intraoperative consumption of sufentanil (Table 1).

**Table 1:** Demographic Data

Characteristics	Group PVB(n = 25)	Group GA(n = 24)	***P*** Value
Age (yr)	58 ± 11	59 ± 9	0.767
Male, n (%)	20 (54)	16 (42)	0.329
BMI	24 ± 3.6	25 ± 3.2	0.126
ASA I/II/III	5/30/2	8/27/3	0.595
Operation time (min)	138 ± 57	129 ± 60	0.571
Estimated blood loss (mL)	33 ± 12	36 ± 13	0.558
Sufentanil dosage (μg)	37 ± 16	68 ± 19	< 0.001
Type of surgery n (%)			0.502
Lobectomy	17 (68)	20 (83)	
Segmentectomy	7 (28)	3 (13)	
Wedge resection	1 (4)	1 (4)	
Histology, n (%)			0.189
Adenocarcinoma	24 (96)	20 (83)	
Squamous	1 (4)	4 (17)	
Others	0 (0)	0 (0)	
Stage, n (%)			0.869
I	21 (84)	19 (79)	
II	3 (12)	3 (13)	
III	1 (4)	2 (8)	
IV	0 (0)	0 (0)	

Values are shown as mean ± standard deviation or number (n) and %. BMI indicates body mass index; ASA, American Society of Anesthesiologists

### Pain scores and consumption of flurbiprofen

**Fig. 2:**
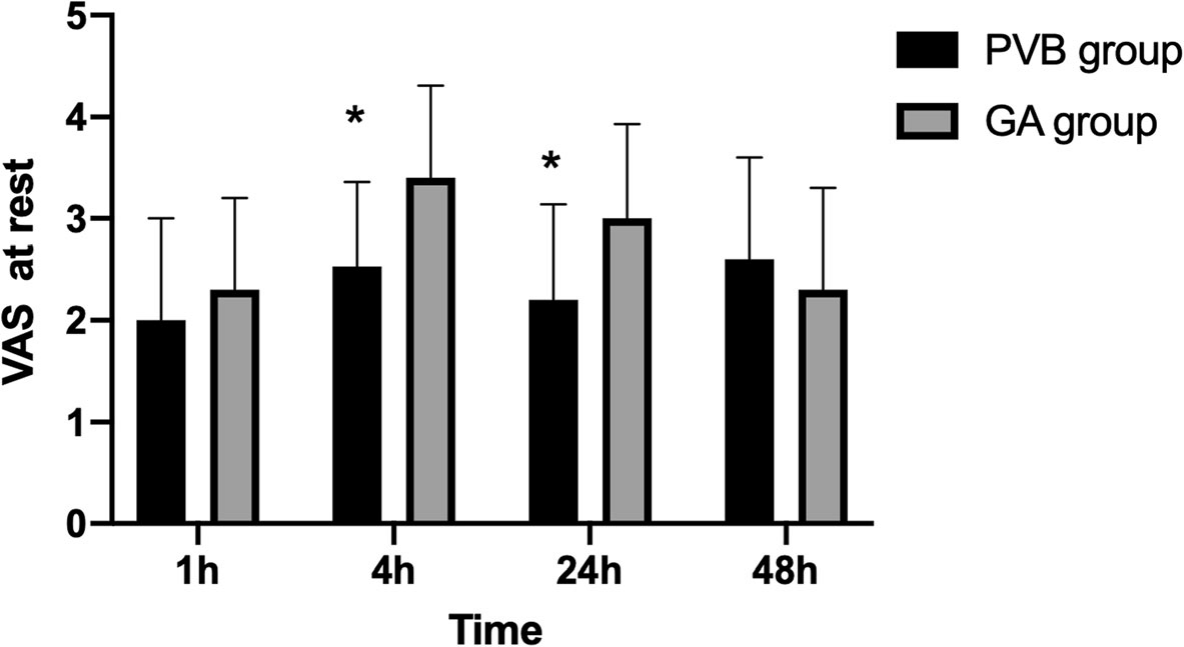
Postoperative pain scores at rest. Pain was assessed by the use of a VAS ranging from 0 to 10 at 1, 4, 24, 48 h after surgery for PVB patients (black bar) and GA patients (gray bar), respectively. VAS scores at rest at 4, 24 h after lobectomy were significantly lower in the PVB group than in the GA group. *Statistical significance (*P* < 0.05). Data are expressed as mean ± standard deviation. VAS = visual analogue scale; PVB = paravertebral block; GA = general anesthesia

**Fig. 3:**
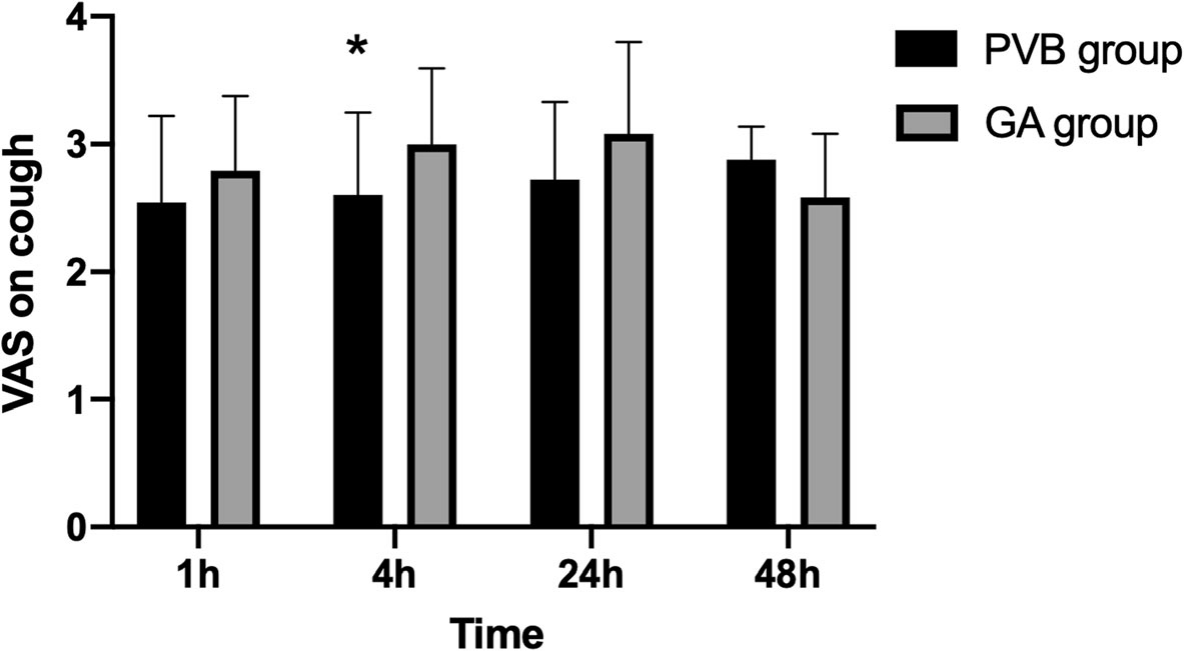
Postoperative pain scores on cough. Pain was assessed by the use of a VAS ranging from 0 to 10 at 1, 4, 24, 48 h after surgery for PVB patients (black bar) and GA patients (gray bar), respectively. VAS scores on cough at 4 h after lobectomy were significantly lower in the PVB group than in the GA group. *Statistical significance (*P* < 0.05). Data are expressed as mean ± standard deviation. VAS = visual analogue scale; PVB = paravertebral block; GA = general anesthesia

VAS pain scores at rest and on cough after VATS lobectomy are shown in Figs. 2 and 3. Compared with the GA group, postoperative VAS pain scores at rest at 4 h [2.53 ± 0.83 (95%CI: 2.20 to 2.86) vs 3.4 ± 0.91 (3.04 to 3.76) respectively, *P* = 0.011] and 24 h [2.2 ± 0.94 (1.83 to 2.57) vs 3.0 ± 0.93 (2.63 to 3.37), *P* = 0.026] were lower in the PVB group. Although there were no difference in VAS on cough at 1 h, 24 h, and 48 h (*P* > 0.05), VAS scores on cough at 4 h was significantly lower in the PVB group than in the GA group (2.6 ±0.65 vs 3.00 ±0.59 respectively, *P* = 0.028). There was no statistically significant difference in VAS scores at rest and on cough between the two groups at 1 h and 48 h after surgery. Total postoperative flurbiprofen consumption was significantly lower in the PVB group compared to GA group. The consumption of flurbiprofen postoperatively was 20 ± 32 mg in the PVB group and 48 ± 43 mg in the GA group respectively, *P* = 0.013.

### Plasma concentrations of MMP-9

**Fig. 4:**
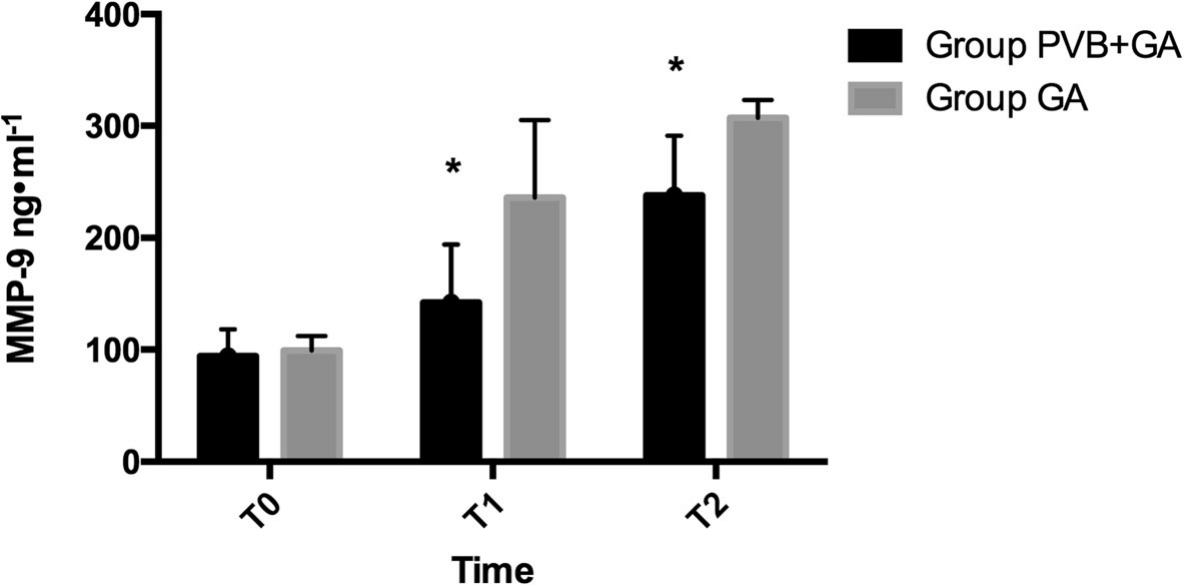
Plasma concentration of measured MMP-9 in lung cancer patients receiving PVB combined general anesthesia or only general anesthesia. *P < 0.05 in the PVB group compared with GA group. MMP-9 = matrix metalloproteinase-9; PVB = paravertebral block; GA = general anesthesia

Mean plasma MMP-9 concentrations at three different time points are shown in Fig. 4. Preoperative MMP-9 did not differ between the PVB group and the GA group [94 ± 24 (95%: 85 to103) vs 99 ± 13 (94 to 104) respectively, *P* = 0.743]. Plasma MMP-9 concentrations increased significantly after surgery compared to preoperative values. Plasma MMP-9 concentrations at T1 and T2 in the PVB group were significantly lower after surgery than in the GA group [142 ± 53 ng/mL(95%CI: 140 to 144) vs 236 ± 69 ng/mL(208 to 264) at T1 respectively, *P* = 0.019; 238 ± 53 ng/mL(95%CI: 217 to 259) vs 307 ± 16 ng/mL(301 to 313) at T2 respectively, *P* = 0.032].

### Complications and length of stay

Postoperative complications are shown in Table 2. Composite complications were uncommon (0–12.5% frequency) and didn’t differ between groups. Although GA group has an increasing trend in postoperative nausea and vomiting (PONV), there was no difference between groups. Mean postoperative hospital stay was not statistically different between the groups (5.3 ± 1.3 days in the PVB group vs. 5.1 ± 1.6 days in the GA group; *P* = 0.647).

**Table 2:** Postoperative complications

Parameter	Group PVB n (%)	Group GA n (%)	*P*-value
Pneumonia	0	1 (4.2)	0.490
Atelectasis	1 (4.0)	2 (8.3)	0.527
Air leak	0	0	
AF	2 (8.0)	1 (4.2)	0.576
Hypotension	3 (12.0)	2 (8.3)	0.672
PONV	1 (4.0)	3 (12.5)	0.277

*AF* atrial fibrillation. *PONV* postoperative nausea and vomiting

## Discussion

Our results showed that VAS pain scores at rest at postoperative 4 h and 24 h and on cough at 4 h were lower in the PVB/GA group. There was no difference in VAS between the groups at other time points. At the same time, plasma MMP-9 levels at the end of surgery and at postoperative 12 h were also significantly decreased in the PVB/GA group after VATS lobectomy. Postoperative complications and the length of hospital stay were not different between the two groups. Although there was an increasing trend in PONV in GA group, there’s no statistics difference in PVB group and GA group.

Surgery is the most effective treatment for lung cancer. However, effective analgesia allows patients to recover quickly. Paravertebral nerve block combined with general anesthesia reduces patients’ immunosuppression and the consumption of sufentanil. Ropivacaine is a long-acting local anesthetic, and its action time can reach 12–24 h. This study showed that patients at the end of surgery had similar VAS scores between groups and that the analgesia effect of paravertebral block was similar to that of sufentanil. Similar pain scores at 1 h were associated with intravenous injection of flurbiprofen at 30 mins before the end of surgery in the both groups. The other reason why VAS at 1 h between the both groups were not difference is that sufentanil has the residual analgesic effect. But the analgesia scores at rest at 4 and 24 h after surgery were lower in the PVB group. The low operative VAS score at 4 h and 24 h in the PVB group may be attributed to the effect of ropivacaine on the paravertebral space for up to 24 h. Analgesia on cough is very important for the patients who have undergone thoracic or upper abdominal surgeries. Our results showed that VAS on cough at 4 h in the PVB group were lower than in the GA group. The reason that PVB patients didn’t have longer analgesia on cough is that we didn’t use continuing PVB analgesia after surgery. If we choose to continue PVB analgesia after lobectomy, it may provide better analgesia when patients were coughing.

Although the tumor is surgically removed, micro-metastasis is inevitable, especially when the patient’s immune function is suppressed. Retrospective studies suggest regional anesthesia including nerve block reduces tumor metastasis and recurrence in various cancers [10–12]. Paravertebral block and postoperative analgesia can reduce the risk of recurrence and metastasis in breast cancer patients during the initial years of follow-up after mammectomy [10].MMP-9 that is a member of the MMP superfamily plays an important role in many pathophysiological processes, such as bone development, wound healing, cell migration, cancer invasion and metastasis [13]. The surgery trauma resulted in increased plasma MMP-9. Our results supported the notion that plasma MMP-9 level increased after VATS lobectomy. PVB inhibited surgical stress and decreased postoperative MMP-9 level. There may be several reasons that PVB decreased plasma MMP-9 level at T1 and T2. First of all, vitro experiments showed local anesthetics have antiproliferative and cytotoxic effects on cancer cells [14–16]. Second, paravertebral nerve block and analgesia reduced the risk of breast cancer recurrence or metastasis 4-fold during a four-year follow-up [10]. Moreover, our observations that reductions in MMP-9 at T1and T2 were greater when patients received combined paravertebral anesthesia with general anesthesia seem consistent with the hypothesis that PVB has little effect on immune function, thus strengthens immune defenses against tumor progression. Thereby, MMP-9 level in the PVB/GA group was lower during VATS lobectomy. Another possible mechanism by which PVB may decrease MMP-9 is that thoracic paravertebral block reduced the level of inflammatory factors and the surgical stress response. Therefore, general anesthesia combined with PVB methods undergoing VATS lobectomy can reduce MMP-9 levels, provide better postoperative analgesia, and should be recommended.

Better analgesia (for example combined PVB) reduces intraoperative consumption of sufentanil. It is well known that opioids can suppress immune function, which may affect tumor metastasis and recurrence. The reasons that opioids promote tumor growth and metastasis are based on the modulation of cellular and humoral responses leading to immunosuppression [17] and the direct action on tumor cells and immune or endothelial cells [18]. The immunosuppressive effect of opioids is independent of their antinociceptive effect. Therefore, it is essential to individually evaluate the effect of opioids on the immune system. During thoracoscopic lobectomy we choosed paravertebral nerve block combined GA so that we can decrease the dose of sufentanil and potentially reduced the inhibition of immune function. If we choose postoperative PVB analgesia, patients in PVB group will have a better recovery.

Although GA group had a high consumption of sufentanil during lobectomy, postoperative complications such as nausea, vomiting, and respiratory depression were not different between the two groups. Previous studies [19, 20] demonstrated the PVB group had a significant reduction in the use of opioids and nerve block can reduce postoperative complications caused by opioids. There could be several reasons for the difference. First, because we did not use PVB as a postoperative analgesia method, the benefits of PVB were not fully shown, such as less pulmonary complications, hypotension, nausea and vomiting, and urinary retention etc. [21]. Second, enhanced recovery after VATS lobectomy protocols we used can prevent factors that delay postoperative recovery and issues that cause complications. Similar results from other studies [22]. Although previous investigations [23] have demonstrated that PVB is associated with shorter hospitalizations, the length of stay was similar in both groups in our study. Hospital stay is affected by various factors, and different postoperative analgesia methods may also affect the length of hospital stay [23].

Our study has several limitations. First, the surgical procedures carried out were not homogeneous. Although the lobectomy is performed by the same group of surgeons, the individual differences and anatomical abnormalities of the surgical patients will cause slight different degrees of surgical injuries. Secondly, postoperative paravertebral analgesia should be adopted in the PVB group, which can better show the difference between the two groups. Third, we should standardize the depth of anesthesia, the time to discharge and use a validated quality of recovery tool such as PosropQRS so that we can better observe the impact of different anesthetic methods on patient recovery. Lastly, we should collect samples of the bio-marker for longer periods to observe the effect of nerve block on the level of MMP-9.

## Conclusions

In conclusion, in this prospective randomized clinical trial, PVB combined general anesthesia is accompanied with an attenuation of MMP-9 response to surgery and provided statistically better pain relief after VATS lobectomy. This technique may be beneficial for patients to recover rapidly after lung surgery and reduce tumor recurrence. Further studies are required to investigate this effect could be extended beyond immediate postoperative period by utilizing a continue paravertebral analgesia technique.

## Data Availability

The data and materials are available from the corresponding author on reasonable request.

## Abbreviations

GA: General anesthesia
MMP-9: Matrix metalloproteinase-9
NSCLC: Non-small cell lung cancer
PACU: Postanesthesia care unit
PCIA: Patient-controlled intravenous analgesia
PONV: Postoperative nausea and vomiting
PVB: Paravertebral block
TPVS: Thoracic paravertebral space
VAS: Visual analog scale
VATS: Video-assisted thoracoscopic surgery
